# Distinct Clinical Characteristics of Pediatric Guillain-Barré Syndrome: A Comparative Study between Children and Adults in Northeast China

**DOI:** 10.1371/journal.pone.0151611

**Published:** 2016-03-14

**Authors:** Xiujuan Wu, Donghui Shen, Ting Li, Bing Zhang, Chunrong Li, Mei Mao, Jixue Zhao, Kangding Liu, Hong-Liang Zhang

**Affiliations:** 1 Neuroscience Center, Department of Neurology, the First Hospital of Jilin University, Jilin University, Changchun, China; 2 Department of Pediatric Surgery, the First Hospital of Jilin University, Jilin University, Changchun, China; Hannover Medical School, GERMANY

## Abstract

**Objective:**

Clinical characteristics of pediatric Guillain-Barré syndrome (GBS) have been extensively studied whereas scarcely been compared with those of adult GBS. Herein we compared the clinical features of GBS between pediatric and adult patients.

**Methods:**

We retrospectively collected the clinical data of 750 patients with GBS (541 adults and 209 children), and compared the clinical characteristics between children and adults.

**Results:**

Pain was a more frequent complaint in children (17.2% vs 9.6%, *p* < 0.01), who were also found with shorter interval from disease onset to nadir (6.3d vs 7.3d, *p* < 0.01) and higher incidence of bulbar dysfunction (22.0% vs 14.8%, *p* < 0.05). The disease severity in children was comparable with adults. In addition, a higher incidence of pediatric GBS was found in summer, especially in July and August (both *p* < 0.01). However, the incidence of antecedent infections of different seasons in adult and pediatric patients was comparable (*p* > 0.05). The clinical features of acute motor axonal neuropathy (AMAN) and acute inflammatory demyelinating polyneuropathy (AIDP) in children were overall comparable with adult ones (*p* > 0.05). Similar to adults, bulbar dysfunction (odds ratio [OR]: 4.621, 95% confidence interval [CI]: 1.240–17.218, *p* < 0.05) and lower nadir Medical Research Council (MRC) sum score (OR: 0.897, 95% CI: 0.855–0.941, *p* < 0.01) were also risk factors for mechanical ventilation in children. However, distinct from adult ones, autonomic dysfunction was significantly higher in mechanically ventilated childhood GBS (39.1% vs 8.8%, *p* < 0.01), which also served as a predictor for mechanical ventilation in pediatric GBS (OR: 70.415, 95% CI: 9.265–535.158, *p* < 0.01). As to the efficacy of intravenous immunoglobulin, insignificant difference was identified between children and adults.

**Conclusion:**

The clinical features of pediatric GBS differ from those of adults. Autonomic dysfunction is an independent risk factor for mechanical ventilation in pediatric patients.

## Introduction

Guillain-Barré syndrome (GBS) is an immune-mediated disorder of the peripheral nervous system which is triggered by either infectious or noninfectious factors [1]. GBS is a predominant cause of acute flaccid paralysis which may occur at any age [2–3]. Clinical features of pediatric GBS have been well characterized in a number of different countries [4–9]. For example, the predominant complaint of the pediatric GBS includes weakness of the limbs, paresthesia, and pain [2]. Autonomic dysfunctions including fluctuating blood pressure, tachyarrhythmia and bradyarrhythmia, abnormal sweating, papillary abnormalities are also common in pediatric GBS [10–11]. In addition, respiratory failure requiring mechanical ventilation is a serious short-term complication of GBS. Previously, we have investigated the clinical predictors for mechanical ventilation in adults with GBS, and we found that shorter interval from disease onset to admission, presence of facial or bulbar dysfunction and lower MRC at nadir were risk factors of mechanical ventilation in adult patients with GBS while disease occurrence in summer was a protective factor [12]. As to the children, symptoms began within 8 days after a preceding infection, cranial nerve involvement, a cerebrospinal protein level > 800 mg/L during the first week, HFGS at nadir, respiratory distress and hypotension were found to serve as predictors for mechanical ventilation [13–14]. Although the clinical characteristics of pediatric GBS have been extensively studied, they were scarcely compared with that of adult ones. In 1994, Sarada and colleagues found that childhood GBS was associated with a higher incidence of cranial nerve palsy and had a more acute form of onset than adults. Moreover, the incidence of respiratory paralysis (40%) and dysautonomia (20%) in children was similar to that in adults [15]. It is noteworthy that the clinical characteristics of GBS in different countries might be distinct due to the geographical diversity and racial difference. Herein we retrospectively investigated the clinical features of pediatric GBS patients and compared the clinical characteristics of GBS between children and adults in northeast China.

## Subjects and Methods

### Subjects

The retrospective study was approved by the ethics committee of the First Hospital of Jilin University, Changchun, China. The records of the recruited patients were anonymized and de-identified prior to analysis. From 2003 to 2014, patients who met the diagnostic criteria of GBS were enrolled [16]. Patients diagnosed as Miller Fisher syndrome, chronic inflammatory demyelinating polyradiculoneuropathy, Bickerstaff encephalitis, or critical illness polyneuropathy/myopathy were ruled out from the study [17]. Moreover, the diagnosis of acute inflammatory demyelinating polyneuropathy (AIDP) and acute motor axonal neuropathy (AMAN) was based on the electrophysiological criteria proposed by Hadden et al. [18]. In our hospital, patients ≤ 16 years old were admitted to the Department of Pediatrics those > 16 years old were admitted to the Department of Neurology. Previously, we have retrospectively investigated the clinical features of adult patients with GBS [12, 19]. The main purpose of this study was to explore the characteristics of pediatric GBS in comparison to adult GBS. Of note is that when comparing the effects of different therapeutic regimens, those who refused any treatments, or those who were discharged within 5 days after admission, or those without available evaluation data of the clinical severity and functional impairment at admission and at discharge, were ruled out from the study.

For all the enrolled patients, clinical parameters including age, sex, preceding infections (mainly the upper respiratory infection and diarrhea), interval from disease onset to admission and time from onset to nadir, functional impairment assessed by the Hughes Functional Grading Scale (HFGS), muscle weakness evaluated by the Medical Research Council (MRC) sum score, sensory disturbances, reflexes, cranial nerve deficits, autonomic dysfunction (e.g. tachyarrhythmia, bradyarrhythmia and abnormal sweating), pain, mechanical ventilation and treatment modality during hospitalization were collected.

### Evaluation of Clinical Severity for Patients with GBS

The clinical severity was evaluated for all the recruited patients. The functional disability was evaluated by the HFGS score which was defined as follows [20]: 0 = healthy state; 1 = minor symptoms and capable of running; 2 = able to walk 5m or more without assistance but unable to run; 3 = able to walk 5m across an open space with help; 4 = bedridden or chair-bound; 5 = requiring assisted ventilation for at least part of the day; 6 = dead. Muscle weakness was assessed by MRC sum score, ranging from 0 (tetraparalytic) to 60 (normal strength) [21]. The nadir of disease was defined as the highest HFGS score or the lowest MRC sum score.

### Treatment and Therapeutic Effect Assessment of Different Treatments

Intravenous immunoglobulin (IVIg) is a first-line option for adult patients with GBS in our hospital due to its safety and convenience. IVIg was usually administered immediately after a clinical diagnosis was established (0.4g/kg/d, for 5 consecutive days). Empirically, intravenous corticosteroids as an add-on therapy were administrated to some of patients whose clinical manifestations deteriorated despite the use of IVIg. If patients refused IVIg or PE (since unaffordable for most of these cases), they usually received either intravenous corticosteroids or supportive treatments. In childhood GBS, 116 patients received IVIg in combination with intravenous corticosteroids, 52 patients received IVIg treatment, and the remaining 43 patients received either intravenous corticosteroids or supportive treatments.

As the hospital duration of patients with or without mechanical ventilation varied markedly, efficacy of IVIg was evaluated by the difference of HFGS and MRC sum score between nadir and 4 weeks after treatment for the mechanically ventilated patients; while for those did not require mechanical ventilation, the therapeutic effect of the treatment was evaluated by the difference of HFGS and MRC sum score between nadir and 2 weeks after treatment. Treatment was considered to be effective if the HFGS score decreased by at least one grade or the MRC sum score was increased by five or more points.

### Statistical Analysis

Statistical analysis was performed with SPSS version 17.0 software (IBM, West Grove, PA, USA). Categorical data were presented as proportions. Continuous data were presented as means and standard deviations or median and interquartile ranges depending on the distribution of the data. Differences in proportions were tested by the Chi-square tests. For continuous variables, Student-*t* test or Mann-Whitney U test were used to compare values between groups. Moreover, independent predictors of mechanical ventilation for pediatric patients with GBS were determined by multivariate logistic regression analysis. For all statistical tests, *p* < 0.05 was considered to be significant.

## Results

### Distinct Clinical Characteristics of Pediatric GBS

A total of 750 patients were enrolled, among whom 541 (72.1%) patients were adults and 209 (27.9%) were children. Comparisons of the clinical characteristics between children and adults were shown in Table 1. Sensory disturbance was more prevalent in adults (50.8% vs 21.5%, *p* < 0.01) while pain was a more common complaint in children (17.2% vs 9.6%, *p* < 0.01). Of note was that higher incidence of facial nerve deficit (31.2% vs 12.0%, *p* < 0.01) was found in adult patients while bulbar dysfunction was a more common complaint in children (22.0% vs 14.8%, *p* < 0.05). With comparable clinical severity, the interval from onset to nadir was shorter in children when compared with adults (6.3d vs 7.3d, *p* < 0.01).

**Table 1:** Comparisons of clinical features of GBS between adults and children.

Variable	Adult (N = 541)	Children (N = 209)	*p*-value
Age	41.6 ± 15.3	9.4±4.5	< 0.01
Male	331 (61.2%)	142 (67.9%)	> 0.05
Time from onset to admission	5.9 (3, 7)	5.5 (3, 7)	> 0.05
Antecedent infections	356 (65.8%)	139 (66.5%)	> 0.05
Hyporeflexia or areflexia	513 (94.8%)	199 (95.2%)	> 0.05
Sensory disturbance	275 (50.8%)	75 (21.5%)	< 0.01
Cranial nerve involvement	237 (43.8%)	69 (33.0%)	< 0.01
Facial nerve	169 (31.2%)	25 (12.0%)	< 0.01
Bulbar dysfunction	80 (14.8%)	46 (22.0%)	< 0.05
Mechanical ventilation	80 (14.8%)	22 (10.5%)	> 0.05
Autonomic dysfunction	27 (5.0%)	15 (7.2%)	> 0.05
Pain	52 (9.6%)	36 (17.2%)	< 0.01
Time from onset to nadir	7.3 (4,9)	6.3 (4, 7)	< 0.01
MRC at nadir	37.4 (28.5, 48.0)	36.2 (30.0, 48.0)	> 0.05
HFGS at nadir	3.3 (3, 4)	3.4 (3, 4)	> 0.05

GBS: Guillain-Barré syndrome; URI: upper respiratory infection; MRC: Medical Research Council

### Seasonal and Monthly Variation in the Occurrence of Pediatric GBS

**Fig 1:**
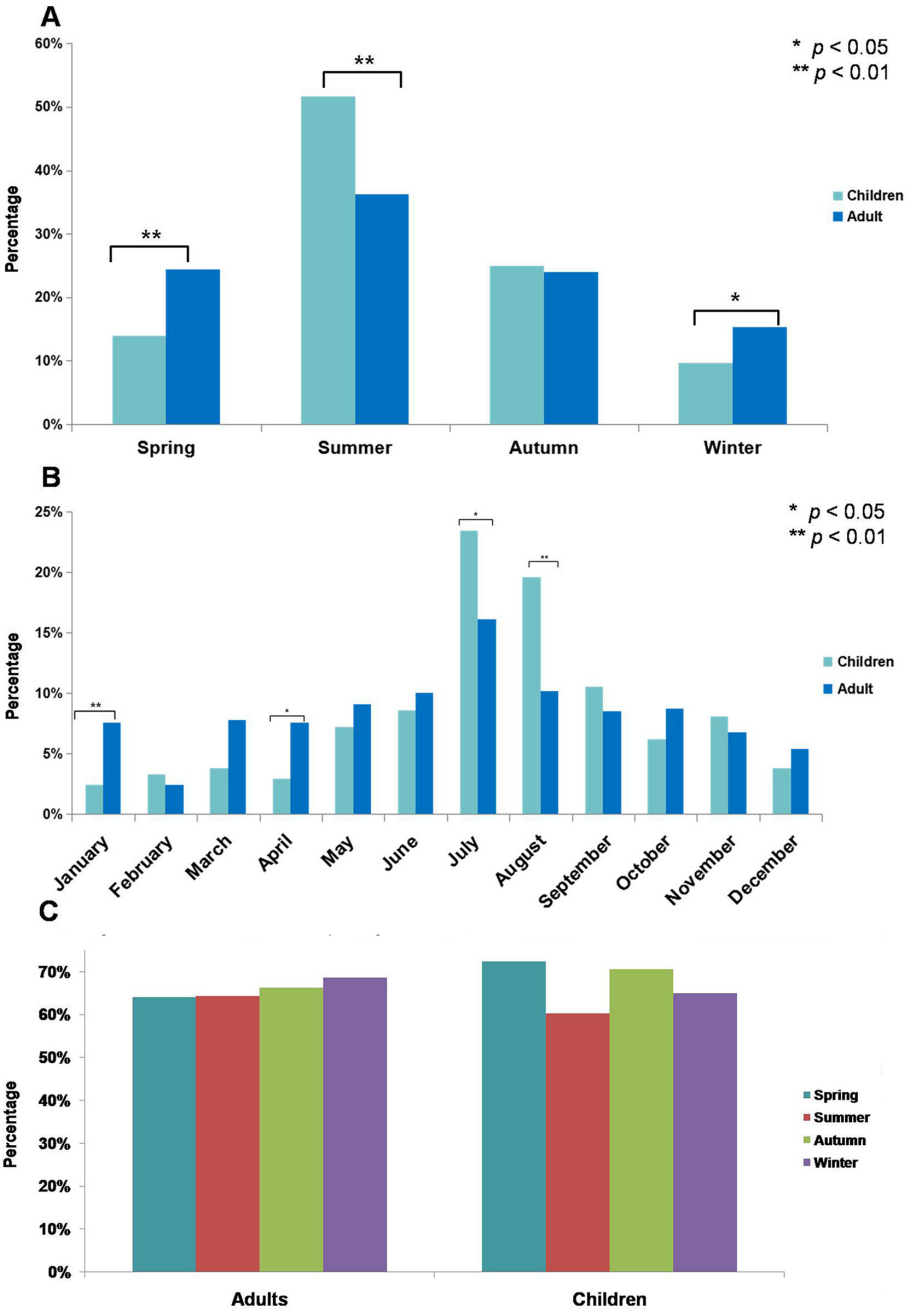
Seasonal and monthly variation in the occurrence of Guillain-Barré syndrome (GBS). The incidence of GBS of pediatric patients in spring, summer, autumn and winter was 13.9%, 51.7%, 24.9% and 9.6%, respectively. Accordingly, it was 24.4%, 36.2%, 24.0% and 15.3%, respectively, in the adult ones (**A**). Further, the monthly variation in the occurrence of GBS was investigated. The incidence of GBS in children and adult during January to December was 2.4% and 7.6%, 3.3% and 2.4%, 3.8% and 7.8%, 2.9% and 7.6%, 7.2% and 9.1%, 8.6% and 10.0%, 23.4% and 16.1%, 19.6% and 10.2%, 10.5% and 8.5%, 6.2% and 8.7%, 8.1% and 6.8%, 3.8% and 5.4%, respectively (**B**). In addition, the incidence of antecedent infections in spring, summer, autumn and winter was 64.0%, 64.3%, 66.3% and 68.6%, respectively, in adults with GBS; similarly, it was 72.4%, 60.2%, 70.6% and 65%, respectively, in pediatric GBS (**C**).

The seasonal and monthly distribution of the adults and children were illustrated in Fig 1. A significantly higher incidence of pediatric GBS was found in summer (51.7% vs 36.2%, *p* < 0.01) when compared with adult ones; while a higher incidence of adult GBS was found in spring (24.4% vs 13.9%, *p* < 0.01) and winter (15.3% vs 9.6%, *p* < 0.05) (Fig 1A). As to monthly variation, incidence of GBS in July and August was significantly higher in children than adults (July: 23.4% vs 16.1%, *p* < 0.05; August: 19.6% vs 10.2%, *p* < 0.01); while it was higher in January and April for adults (January: 7.6% vs 2.4%, *p* < 0.01; April: 7.6% vs 2.9%, *p* < 0.05) (Fig 1B). Further the incidence of antecedent infections in different seasons was compared (Fig 1C). The incidence of antecedent infections in different seasons in children did not differ from that in adults (*p* > 0.05).

### Comparisons of Clinical Features between AMAN and AIDP

The results of the electrophysiological examination during hospitalization were available in 373 patients, and 286 patients were adults while 87 were children. AIDP was identified in 120 patients (92 adults and 28 children) while AMAN was identified in 68 patients (45 adults and 23 children). Additionally, the electrophysiological examination of 143 patients was equivocal while 42 patients were normal. As shown in Table 2, the clinical characteristics and disease severity of AIDP between children and adults were overall comparable. Similarly, the clinical features of pediatric AMAN did not differ from those of adults (data not shown).

**Table 2:** Comparisons of clinical features of AIDP between adults and children.

Variable	Adult (N = 92)	Children (N = 28)	*p*-value
Age	43.0 ± 15.6	11.1 ± 3.7	
Male	57 (62.0%)	18 (64.3%)	> 0.05
Time from onset to admission	7.33 (3.0, 10.0)	6.2 ± 3.4	> 0.05
Antecedent infections			
URI	29 (31.5%)	8 (28.6%)	> 0.05
Diarrhea	33 (35.9%)	12 (42.9%)	> 0.05
Cranial nerve involvement			
Facial nerve	26 (28.3%)	4 (14.3%)	> 0.05
Bulbar dysfunction	10 (10.9%)	2 (7.1%)	> 0.05
Mechanical ventilation	6 (6.5%)	0	> 0.05
Autonomic dysfunction	2 (2.2%)	0	> 0.05
Pain	8 (8.7%)	8 (28.6%)	< 0.05
Time from onset to nadir	8.7 (5.0, 11.8)	7.0 ± 3.0	> 0.05
MRC at nadir	41.3 (35.3, 50.0)	39.8 ± 11.0	> 0.05
HFGS at nadir	3.1 (2.0, 4.0)	3.4 (3.0, 4.0)	> 0.05

AIDP: acute inflammatory demyelinating polyneuropathy; URI: upper respiratory infection; MRC: Medical Research Council; HFGS: Hughes Functional Grading Scale

**Fig 2:**
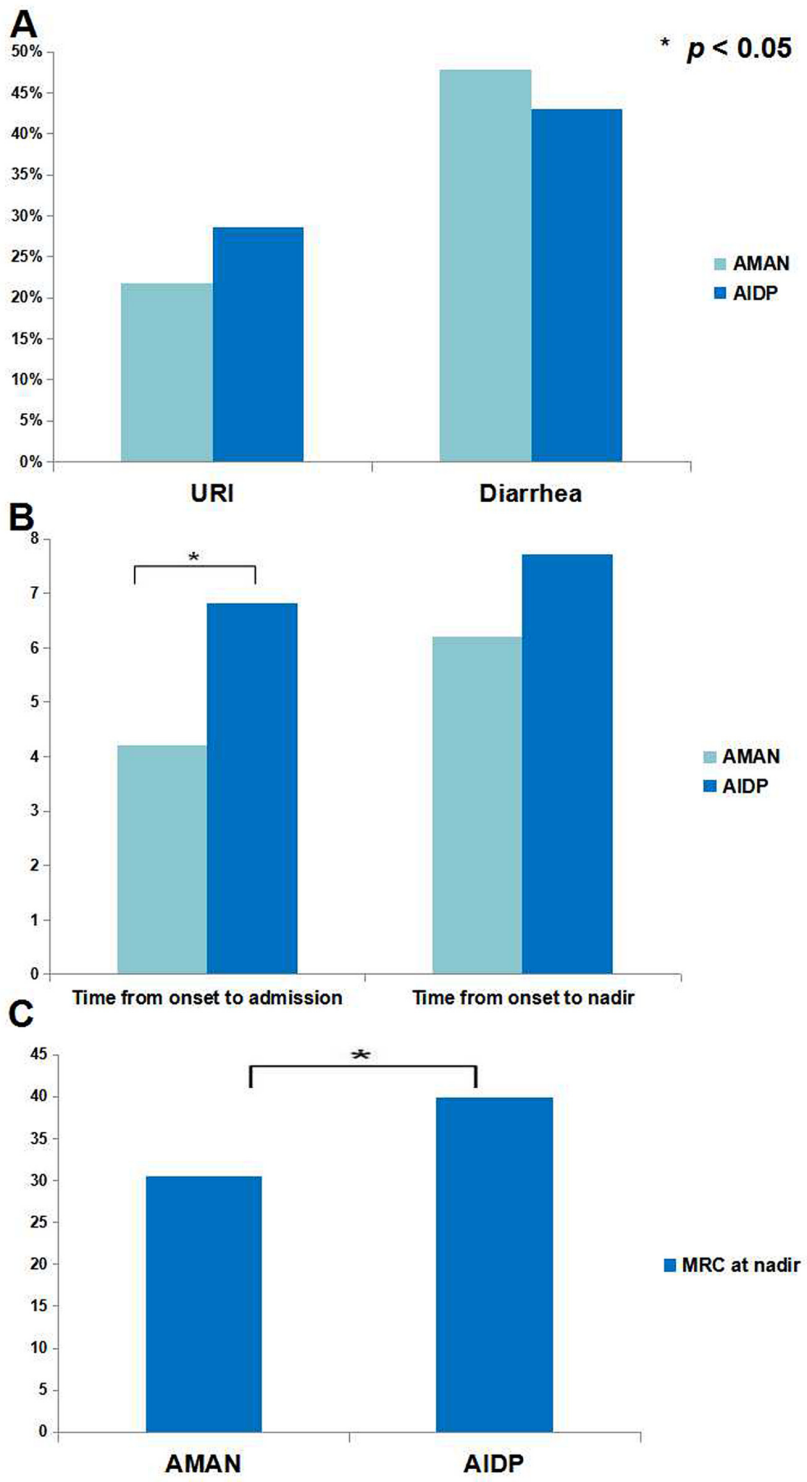
Comparisons between AMAN and AIDP in children. The incidence of upper respiratory infection (URI) as antecedent infections of AMAN was 21.7% which was insignificantly different from AIDP (28.6%, *p* > 0.05). Similarly, the incidence of diarrhea in pediatric AMAN was comparable with AIDP (47.8% vs 42.9%, *p* > 0.05) (**A**). The interval from onset to admission was 4.2d in children with AMAN, while it was 6.2d for pediatric AIDP, which was significantly different. However, interval from onset to nadir was comparable between AMAN and AIDP (6.2d vs 7.0d, *p* > 0.05) (**B**). The MRC sum score at nadir was lower in pediatric AMAN than childhood AIDP (30.5 ±12.0 vs 39.8±11.0, *p* < 0.05) (**C**). AMAN: acute motor axonal neuropathy; AIDP: acute inflammatory demyelinating polyneuropathy; URI: upper respiratory infection; MRC: Medical Research Council.

We further compared the clinical characteristics of AIDP with AMAN in children. The proportion of male was significantly higher in AMAN than AIDP (91.3% vs 64.3%, *p* < 0.05); however, incidence of antecedent infections was comparable (URI: 21.7% vs 28.6%; diarrhea: 47.8% vs 42.9%; both *p* > 0.05) (Fig 2A). Incidence of facial nerve palsy (26.1% vs 14.3%, *p* > 0.05) and bulbar dysfunction (26.1% vs 7.1%, *p* > 0.05) in AMAN was not different from AIDP. The interval from onset to admission was shorter in AMAN than AIDP (4.2±3.3 vs 6.2±3.4, *p* < 0.05) while interval from onset to nadir was comparable (6.2±4.1 vs 7.0±3.0, *p* > 0.05) (Fig 2B). The nadir MRC was lower in AMAN than AIDP (30.5 ±12.0 vs 39.8±11.0, *p* < 0.05) (Fig 2C) while the HFGS at nadir was comparable (3.9 vs 3.4, *p* > 0.05), indicating that motor impairment was more severe in pediatric AMAN.

### Predictors for Mechanical Ventilation in Pediatric GBS

A total of 103 patients were mechanically ventilated during hospitalization, including 80 adults and 23 children. The clinical predictors for mechanical ventilation in pediatric GBS were explored. Univariate analysis revealed that interval from onset to admission (OR: 0.686, 95% CI: 0.529–0.889, *p* < 0.01), facial nerve palsy (OR: 5.681, 95% CI: 1.720–10.681, *p* < 0.01), bulbar dysfunction (OR: 4.286, 95% CI: 2.086–15.467, *p* < 0.01), autonomic dysfunction (OR: 50.833, 95% CI: 12.333–209.515, *p* < 0.01), and MRC at nadir (OR: 0.903, 95% CI: 0.871–0.936, *p* < 0.01) were statistically significant parameters. As illustrated in Table 3, bulbar dysfunction, autonomic dysfunction, and a lower nadir MRC were risk factors for mechanical ventilator in pediatric GBS. Of note was that autonomic dysfunction was an independent risk factor for mechanical ventilation in pediatric patients, which was distinct from adults. This was further supported by the comparisons of clinical features between mechanically ventilated children and adults (Table 4).

**Table 3:** Independent predictors for mechanical ventilation in children with GBS.

Variable	Regression coefficient (95% CI)	*p*-value	Exp (B)
Bulbar dysfunction	1.531 (1.240–17.218)	< 0.05	4.621
Autonomic dysfunction	4.254 (9.265–525.158)	< 0.01	70.415
MRC at nadir	-0.109 (0.855–0.941)	< 0.01	0.897

GBS: Guillain-Barré syndrome; MRC: Medical Research Council; CI: confidence interval

**Table 4:** Comparisons of the clinical characteristic of mechanically ventilated patients with GBS between adults and children.

Variable	Adult (N = 80)	Children (N = 23)	*p*-value
Age (year-old)	43.8 ± 16.7	10.5±5.0	
Male	51 (63.8%)	17 (73.9%)	> 0.05
Antecedent infections	55 (68.75%)	17 (73.9%)	> 0.05
URI	32 (40%)	11 (47.8%)	> 0.05
Diarrhea	22 (27.5%)	4 (17.4%)	> 0.05
Time from onset to admission	3.5 ± 3.2	2.9 ± 1.8	> 0.05
Cranial nerve involvement	53 (66.25%)	16 (69.6%)	> 0.05
Facial nerve	43 (53.8%)	8 (34.8%)	> 0.05
Bulbar dysfunction	30 (56.6%)	11 (47.8%)	> 0.05
Autonomic dysfunction	7 (8.8%)	9 (39.1%)	< 0.01
Pain	7 (8.8%)	1 (4.3%)	> 0.05
Time from onset to nadir	5.9 ± 3.6	6.1 ± 3.9	> 0.05
MRC at nadir	16.6 ± 16.6	13.1 ± 14.5	> 0.05

GBS: Guillain-Barré syndrome; URI: upper respiratory infection; MRC: Medical Research Council

### Comparable Efficacy of IVIg between Pediatric and Adult GBS

In total, 255 adult patients receiving IVIg; however, only 246 patients enrolled in the study, because 9 patients discharged within 5 days and therapeutic effect assessment was not available for these patients. The efficacy of IVIg was comparable between children and adults (Table 5). In patients receiving combination therapy, the clinical severity and dose of the intravenous corticosteroids were largely different between pediatric and adult patients (Table 6). Thus the efficacy of IVIg combined with intravenous corticosteroids was not compared between children and adults.

**Table 5:** Comparisons of therapeutic effect of IVIg between children and adults.

Variable	Adults(N = 246)	Children(N = 52)	*p*-value
Mean age (year-old)	41.9±16.0	10.9±4.6	
Ratio of male	4.8±3.0d	31 (59.6%)	> 0.05
Time from onset to admission	6.4±4.0d	4.9 (3.0, 5.0)	> 0.05
Time from onset to nadir	164 (66.7%)	5.6 (3.0, 7.0)	> 0.05
Antecedent infections	114 (46.3%)	36 (69.2%)	> 0.05
Sensory disturbance	93 (37.8%)	18 (34.6%)	> 0.05
Cranial nerve involvement	227 (92.3%)	18 (34.6%)	> 0.05
Mechanical ventilation	33 (13.4%)	5 (9.6%)	> 0.05
Proportion of severe GBS by MRC (≤ 36 points)	125 (50.8%)	28 (53.8%)	> 0.05
Proportion of severe GBS by HFGS (> 3 points)	148 (60.2%)	30 (57.7%)	> 0.05
MRC sum score at nadir	35.4±20.0	34.8 (24.0, 45.8)	> 0.05
HFGS at nadir	3.5±1.0	3.6 (3.0, 4.0)	> 0.05
Effective rate of the therapy assessed by MRC	184 (74.8%)	45 (86.5%)	> 0.05
Effective rate of the therapy assessed by HFGS	162 (65.9%)	38 (73.1%)	> 0.05

IVIg: intravenous immunoglobulin; HFGS: the Hughes Functional Grading Scale; MRC: Medical Research Council

**Table 6:** Comparisons of clinical characteristics between pediatric and adult GBS receiving IVIg combined with intravenous corticosteroids.

Variable	Adults(N = 101)	Children(N = 114)	*p*-value
Mean age (year-old)	40.8±17.0	8.5±4.3	
Ratio of male	69 (68.3%)	80 (70.2%)	> 0.05
Time from onset to admission (d)	4.3±3.0d	5.0 (2.8, 7.0)	< 0.05
Time from onset to nadir (d)	7.0±5.0d	6.3 (4.0, 7.0)	> 0.05
Antecedent infections	65 (64.4%)	69 (60.5%)	> 0.05
Sensory disturbance	60 (59.4%)	41 (36.0%)	< 0.01
Cranial nerve involvement	56 (55.4%)	35 (30.7%)	< 0.01
Mechanical ventilation	95 (94.1%)	9 (7.9%)	< 0.01
Proportion of severe GBS by MRC (≤ 36 points)	64 (63.4%)	55 (48.2%)	> 0.05
Proportion of severe GBS by HFGS (> 3 points)	81 (80.2%)	64 (56.1%)	< 0.01
MRC sum score at nadir	28.8±30.0	35.6 (28.5, 48.0)	< 0.01
HFGS at nadir	3.9±2.0	3.5 (3.0, 4.0)	< 0.01

GBS: Guillain-Barré syndrome; IVIg: intravenous immunoglobulin; HFGS: the Hughes Functional Grading Scale; MRC: Medical Research Council

## Discussion

In this study, we found that the clinical characteristics in pediatric GBS differed from that in adult ones. However, the clinical features of AIDP and AMAN which were different from each other in children, were overall comparable between children and adults. Autonomic dysfunction was an independent risk factor for mechanical ventilation in pediatric GBS, which was distinct from adult patients. As to the therapeutic effect of IVIg, we found it was comparable between children and adults.

The clinical characteristics of GBS in children from different studies are not consistent, which might be due to geographical and racial diversity [4–9, 22–24]. In this study, we found that the clinical characteristic in pediatric GBS differed from that in adult ones. Sensory disturbance was more prevalent in adults while pain was a more common complaint in children. The incidence of pain in our study was in consistence with the study by Hung et al [23]. Our reported incidence of pain might be lower than the actual proportion in that some children patients could not describe their symptoms. The incidence of facial nerve deficit in children was lower than adults. By contrast, the incidence of bulbar dysfunction in children was higher than that in adults. Seasonal or monthly variation in the occurrence of GBS was also found in our study. The seasonal and monthly variation in the occurrence of GBS from different studies remains controversial [23, 25–27]. The reasons for this disparity remain unclear and still need further elucidation. In our study, a higher incidence of GBS was found in summer both for the children and adults, especially in July and August. This trend was more obvious in pediatric patients. Our finding was in accordance with the study by Paradiso and colleagues [25]. However, this seasonal predominance could not be attributed to the higher incidence of antecedent infections, because we found that the incidence of antecedent infections in different seasons was comparable. It is noteworthy that the microbiology data was not available due to the retrospective nature of our study. Whether this seasonal variation correlates to different pathogens, which might be asymptomatic or lead to non-specific symptoms, warrants further elucidation.

AIDP and AMAN are major subtypes of GBS, and they are distinct from each other in terms of clinical characteristics, immunopathogenesis, electrophysiological findings, pathological changes and responses to treatment [28]. We found that the clinical features of AMAN and AIDP in children were overall comparable with adults. However, we found that the characteristics of pediatric AIDP were different from childhood AMAN. The proportion of male was higher in pediatric AMAN, and this finding was contradictory to the study by Nachamkin and colleagues [29]. Their study was based on a Mexican population, and they found that the male to female ratio was 1.3 for AMAN cases while it was 3.0 for AIDP cases. Moreover, the motor impairment revealed by the nadir MRC sum score was significantly higher in pediatric AMAN, which was associated with shorter interval from onset to admission, indicating an acute onset and a more severe clinical severity of pediatric AMAN. The finding was in agreement with a previous study [29]. However, another study based on the Chinese children revealed no significant difference in the functional status at nadir between AMAN and AIDP [9]. Due to the small sample size, the comparisons between AIDP and AMAN in children, as well as that between children and adults, need further study to validate.

Respiratory failure requiring mechanical ventilation is a common and serious short-term complication of GBS, and the incidence was variable in pediatric patients from different studies [6–7, 9, 13–14]. In our study, bulbar dysfunction and a lower nadir MRC were risk factors for mechanical ventilator in pediatric GBS, which was similar to adult ones. Of note was that autonomic dysfunction also served as an independent risk factor for pediatric patients requiring mechanical ventilator. Thus, children with GBS who were found with autonomic dysfunction during hospitalization should pay more attention due to their increased risk for respiratory failure. This finding is warranted to further validation.

There are limitations of our study. Due to the retrospective nature of the study and the failure to make follows-up on patients, the current sample size is too small for the stratified analysis like comparisons of the clinical characteristic between pediatric AMAN and AIDP, as well as the comparisons of different subtypes of GBS between children and adults. Besides, the electrophysiological results were equivocal for a number of patients, which was higher than previous studies. As AMAN was more common in Asia, and the electrophysiological findings of AMAN at the very early stage might be reversible conduction failure (RCF), which is characteristic of AIDP and needs to be reevaluated by serial recordings [30]. However, we failed to obtain the electrophysiological data during follows-up. Moreover, the prognostic factors for mechanically ventilated children were not investigated due to the small sample size.

In summary, the clinical characteristics in pediatric GBS were different from that in adults, including the accompanying symptoms, seasonal and monthly variation, predictors of mechanical ventilation. Similar to adults, bulbar dysfunction and a lower nadir MRC were risk factors for mechanical ventilation in pediatric GBS. Of note was that autonomic dysfunction was an independent risk factor for mechanically ventilated children, which was distinct from adults.
